# Tetralogy of Fallot with vertebral defect and left aberrant subclavian artery: a rare occurrence

**DOI:** 10.1186/s43044-024-00454-w

**Published:** 2024-02-20

**Authors:** Marya Hameed, Muhammad Fazal Hussain Qureshi

**Affiliations:** Department of Radiology, National Institute of Child Health, Karachi, Pakistan

**Keywords:** Tetralogy, Septal defect, Artery, Cyanosis, Rare

## Abstract

**Background:**

The most prevalent cyanotic congenital heart disease is Tetralogy of Fallot (TOF). It has a variety of presentations and is made up of four anatomic abnormalities. Documented literature shows an incidence of 13–20% of a right aortic arch with an anomalous left subclavian artery among individuals diagnosed with TOF. This is the first case that discusses the rare occurrence of overriding of the aortic arch along with the left aberrant subclavian artery and vertebral defect in a 3-week-old girl. Timely identification and management are pivotal in ensuring the best possible outcomes for these young patients.

**Case presentation:**

A 3-week-old female child came with complaints of dyspnea, dysphagia, fatigue, and cyanosis on extreme crying, feeding, and moderate activity. Echocardiography revealed severe pulmonary stenosis with right ventricular dilatation and ventricular septal defect (VSD); a chest computed tomography was performed that revealed four characteristic features of TOF (pulmonary artery stenosis, VSD, right aortic root deviation, and concentric right ventricular hypertrophy) along with overriding of the aortic arch accompanied with the left aberrant subclavian artery (compressing the trachea and infundibulum) and vertebral defect (butterfly vertebra).

**Conclusions:**

The case of this 3-week-old female infant emphasizes the importance of early and accurate diagnosis in congenital heart diseases, particularly when faced with complex presentations such as the TOF. It serves as a testament to the valuable role of advanced diagnostic imaging techniques in unraveling the complexity of congenital heart conditions.

## Background

The most prevalent cyanotic congenital heart disease is the Tetralogy of Fallot (TOF). It has a variety of presentations and is made up of four anatomic abnormalities, including pulmonary artery stenosis, intraventricular communication, right aortic root deviation, and concentric right ventricular hypertrophy. TOF affects males and females equally, occurring in 3 to 5 neonates in every 10,000 live births [1]. The extent of obstruction in the right ventricular outflow tract greatly influences the timing when symptoms become apparent. The pathophysiological mechanism behind Tetralogy of Fallot (TOF) involves the redirection of deoxygenated blood from the systemic veins through the ventricular septal defect, leading to its mixing with oxygenated systemic blood. The identification of TOF instances is of utmost importance due to their significant potential for successful recovery subsequent to repair [2]. According to the existing literature, there is a documented incidence of 13–20% of a right aortic arch with an anomalous left subclavian artery among individuals diagnosed with Tetralogy of Fallot (TOF) [3]. Literature regarding this topic is still scarce, and this is the first case that discusses the rare occurrence of overriding of the aortic arch along with the left aberrant subclavian artery and vertebral defect in a 3-week-old girl.

## Case presentation

A 3-week-old female infant came with complaints of dyspnea, dysphagia, fatigue, and cyanosis on extreme crying, feeding, and moderate activity. The family history records did not point toward any history of congenital heart disease. She was a full-term baby, with a weight at birth of 2500 g and an APGAR score of 8. Clinical examination revealed cold and pale extremities with discreet perioral cyanosis. The apex beat was present in the 5th intercostal space on the middle-clavicular line, pulse rate = 100/min, BP = 100/60 mm Hg, rhythmic heartbeats; a rough systolic ejection murmur was heard at the left upper sternal border due to pulmonic stenosis and holosystolic murmur at the left mid sternal border indicating toward a ventricular septal defect. Echocardiography revealed severe pulmonary stenosis with right ventricular dilatation and ventricular septal defect (VSD).

**Fig. 1:**
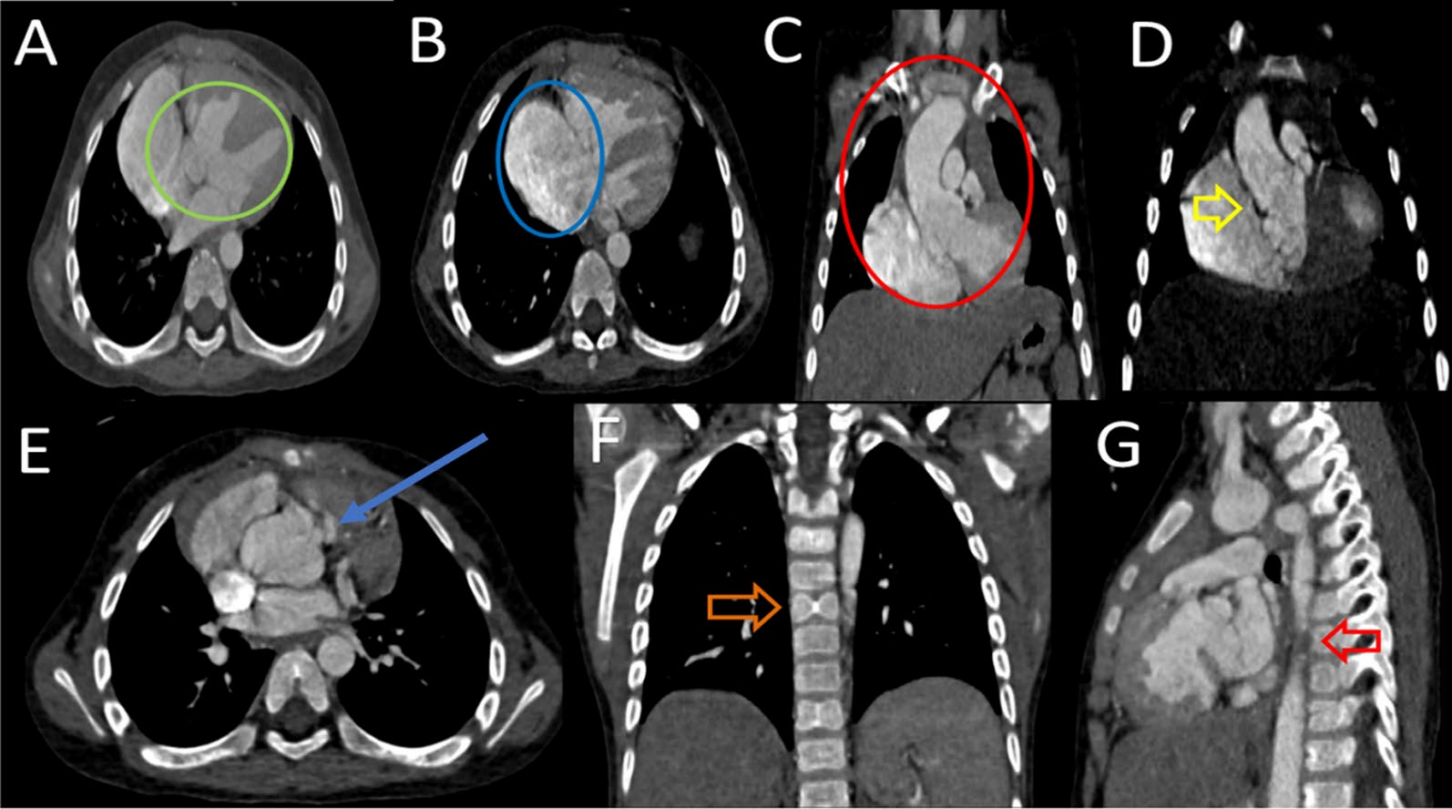
**A** A large VSD noted in the membranous part (green circle), **B** right chamber dilation (blue circle), **C** overriding of aorta with right chamber dilatation (red circle), **D** Right outflow tract narrowing (yellow arrow), **E** pulmonary stenosis (blue arrow), **F** Butterfly vertebra (orange arrow), **G** vertebral segmentation and infundibular narrowing (red arrow)

**Fig. 2:**
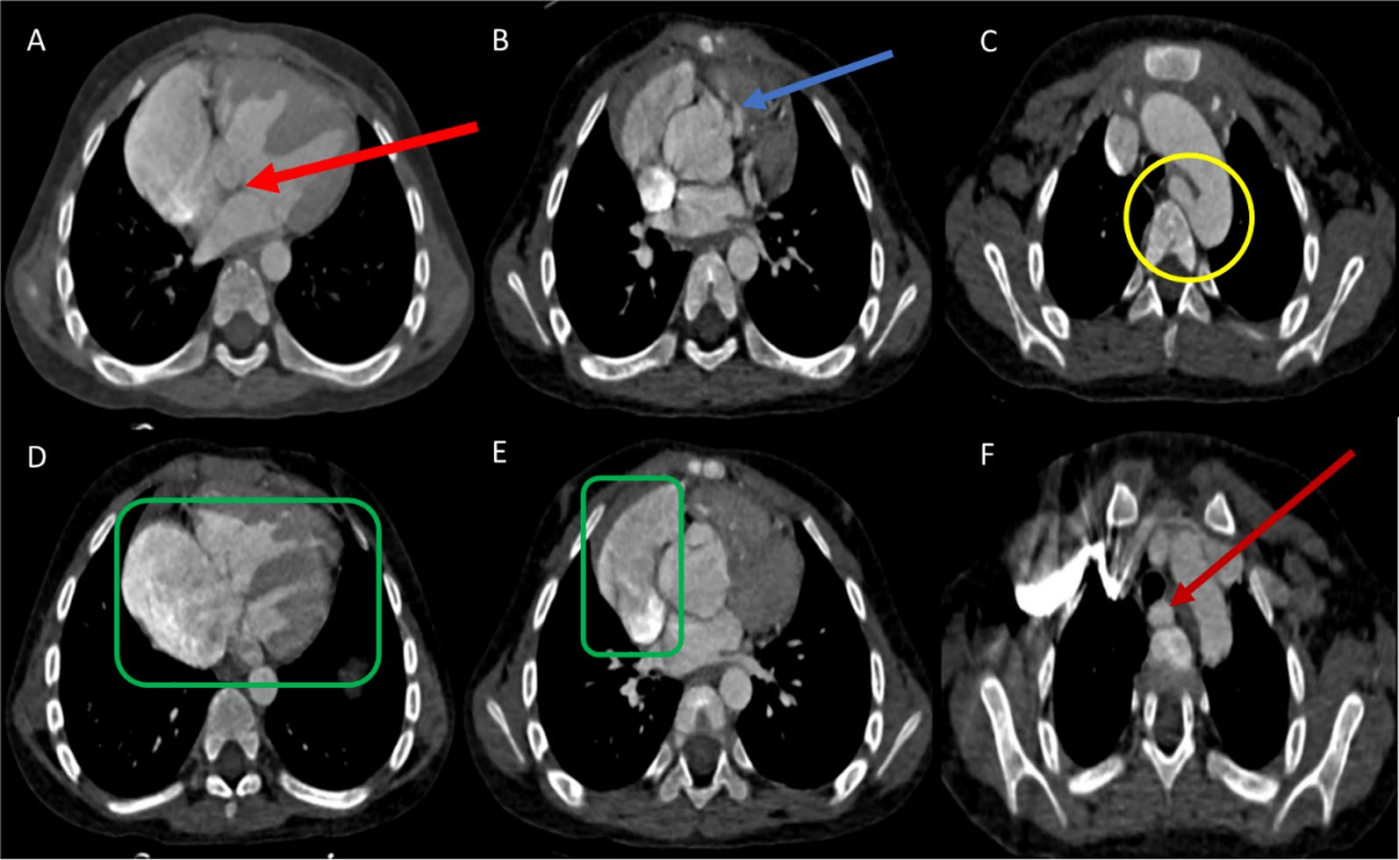
**A** Ventricular septal defect (red arrow), **B** Pulmonary Stenosis (Blue arrow), **C**, **F** Left aberrant subclavian artery (yellow circle and red arrow), **D**, **E** Right chamber dilatation and papillary muscle hypertrophy (green rectangle)

A chest computed tomography was performed that revealed four characteristic features of TOF (pulmonary artery stenosis, ventricular septal defect, right aortic root deviation, and concentric right ventricular hypertrophy) along with overriding of the aortic arch accompanied with the left aberrant subclavian artery (compressing the trachea and infundibulum) and vertebral defect (butterfly vertebra) as shown in Figs. 1 and 2.

## Discussion

This case exemplifies the intricate nature of TOF, which can manifest in diverse ways, posing a diagnostic challenge. The co-occurrence of several anomalies, as highlighted in this case, underscores the importance of comprehensive imaging techniques, such as echocardiography and chest CT, to provide a holistic understanding of the condition.

Furthermore, the absence of a family history of congenital heart disease serves as a reminder that these conditions can occur in isolation without prior familial predisposition. The case also highlights the need for a high index of suspicion in cases with unusual or atypical symptoms, especially in infants and neonates.

The right aortic arch is an infrequent congenital anomaly, with an estimated occurrence rate of roughly 0.1%. This condition is characterized by the positioning of the aortic arch on the right side of the trachea. This developmental aberration manifests during the period of embryogenesis spanning from weeks 4 to 5. It is characterized by the migration and formation of the six pairs of aortic arch arteries, which connect the ventral aorta to both dorsal aortae, resulting in the development of their respective structures [4].

The prevalence of an anomalous left subclavian artery in persons with a right aortic arch is estimated to be around 0.04–0.1% based on observations from postmortem specimens. Radiological examinations have yielded similar findings, with a reported incidence of approximately 0.05% [5]. Moreover, there have been a limited number of reports documenting its correlation with an uncommon anomaly known as the congenital absence of the left pulmonary artery [6]. Individuals who possess a right aortic arch with an anomalous left subclavian artery frequently exhibit no symptoms. Nevertheless, it is typically the symptoms related to the compression of neighboring structures, such as the esophagus and trachea, that provide indications of concomitant vascular anomalies in conjunction with tetralogy of Fallot (TOF) [7].

The utilization of chest radiography is advantageous in the evaluation of the right aortic arch, while barium enema can be employed for the identification of esophageal compression. The identification of an anomalous left subclavian artery is confirmed through the utilization of medical imaging techniques such as CT scan, MRI, or 2D echocardiography with color Doppler [8, 9]. Patients presenting with a right aortic arch and an abnormal subclavian artery necessitate surgical surgery.

## Conclusion

To summarize, the instance of this 3-week-old female newborn highlights the crucial importance of prompt and accurate identification in congenital heart conditions, particularly when complex symptoms such as Tetralogy of Fallot are present. This instance exemplifies the crucial significance of sophisticated diagnostic imaging tools in unraveling the intricacies of congenital heart diseases. Timely recognition and treatment of these disorders are crucial for achieving the best results in these young patients, highlighting the essential role of interdisciplinary teams and advanced medical technologies in the field of pediatric cardiology.

## Data Availability

All the data regarding this case are a part of this manuscript.

## Abbreviations

TOF: Tetralogy of Fallot
CT: Computed tomography
MRI: Magnetic resonance imaging
VSD: Ventricular septal defect
